# Ethical Implications of the Use of Language Analysis Technologies for the Diagnosis and Prediction of Psychiatric Disorders

**DOI:** 10.2196/41014

**Published:** 2022-11-01

**Authors:** Alexandre Andrade Loch, Ana Caroline Lopes-Rocha, Anderson Ara, João Medrado Gondim, Guillermo A Cecchi, Cheryl Mary Corcoran, Natália Bezerra Mota, Felipe C Argolo

**Affiliations:** 1 Institute of Psychiatry University of Sao Paulo Sao Paulo Brazil; 2 Instituto Nacional de Biomarcadores em Neuropsiquiatria Conselho Nacional de Desenvolvimento Científico e Tecnológico Brazilia Brazil; 3 Departamento de Estatística Universidade Federal do Paraná Curitiba Brazil; 4 Instituto de Computação Universidade Federal da Bahia Salvador Brazil; 5 IBM Thomas J. Watson Research Center Yorktown Heights, NY United States; 6 Icahn School of Medicine at Mount Sinai New York, NY United States; 7 Instituto de Psiquiatria Universidade Federal do Rio de Janeiro Rio de Janeiro Brazil; 8 Research Department at Motrix Lab Motrix Rio de Janeiro Brazil

**Keywords:** at-risk mental state, psychosis, clinical high risk, digital phenotyping, machine learning, artificial intelligence, natural language processing

## Abstract

Recent developments in artificial intelligence technologies have come to a point where machine learning algorithms can infer mental status based on someone’s photos and texts posted on social media. More than that, these algorithms are able to predict, with a reasonable degree of accuracy, future mental illness. They potentially represent an important advance in mental health care for preventive and early diagnosis initiatives, and for aiding professionals in the follow-up and prognosis of their patients. However, important issues call for major caution in the use of such technologies, namely, privacy and the stigma related to mental disorders. In this paper, we discuss the bioethical implications of using such technologies to diagnose and predict future mental illness, given the current scenario of swiftly growing technologies that analyze human language and the online availability of personal information given by social media. We also suggest future directions to be taken to minimize the misuse of such important technologies.

## Introduction

In 2018, football commentator and former Liverpool defender Mark Lawrenson was alerted to a facial cancerous blemish by one of his viewers [1]. General practitioner Alan Brennan emailed England’s BBC and alerted Lawrenson after watching him on TV and spotting the suspicious skin lesion. Lawrenson successfully treated the skin cancer and later would bring the doctor to the TV show to interview and thank him. In 2020, reporter Victoria Price was on air when a spectator noticed a lump on her neck [2]. The woman promptly emailed the reporter alerting that Price should have her thyroid checked—the spectator reported she also had a neck bulging in the past that was revealed to be cancer. After exams and appointments with oncologists, Price confirmed that the lump was a thyroid cancer and underwent an effective treatment [2]. These are two examples among many others in which signs of someone’s undiagnosed disease could be noticed by a third party who seized the opportunity to alert the person about it. Such attitudes are often lifesaving, as they end up in diagnosis and effective treatment. However, what if mental illness could somehow also become perceptible?

In this paper, we discuss the intrinsic privacy protection of mental illnesses and how current technologies, specifically artificial intelligence (AI), allow us to “see” mental illness and potentially bypass this protection. By “see,” we mean to view by digital means. Stigma is then addressed, as it is the main issue that makes the ability to “see” mental illness have different consequences as compared to “seeing” other illnesses. Bioethical issues related to both previous items and to the use of such technologies are discussed. We then address the interpretability of AI models, an issue that may threaten bioethical principles. Lastly, we discuss problems related to the use of such technologies outside clinical and research settings.

## The Privacy Protection of Mental Illness

We usually think of psychiatric illness as having an intrinsic privacy protection, since we need someone to talk about their thoughts and feelings to make a mental status assessment. However, many psychiatric disorders are somewhat apparent to the trained eye—and ears—of the mental health professional and even to lay people. Appearance and behavior are the first items in the mental status examination, a road map for mental health professionals that is equivalent to the physical exam of the general practitioner [3]. Along this examination, the content and form of what someone says is also carefully assessed, as speech is the main access we have to the patient’s thoughts and feelings. These are all items of what we denote in a broad definition as communication: the first is called nonverbal communication (or nonverbal language), and the other is verbal communication [4]. Therefore, the way someone behaves and what someone says, even while not being in a psychiatric interview, can sometimes provide enough data to presume the possibility of a mental disorder. This is similar to the case with jaundice, weight loss, or lumps across the body in other illnesses, for instance. However, confirmation of the diagnosis is dependent upon further examination beyond the signs shown. This confirmation, as well as the disclosure of the diagnosis, is contingent on the patient’s acquiescence to be submitted to laboratory tests, imaging, and physical examination, and to reveal their feelings and experiences by further questioning about their mental status during an interview. Nevertheless, this “privacy protection” might be overcome by technology and by the quick and recent progress in AI modeling.

To begin with, the wide use of social media has made an unprecedented amount of private data publicly available. This is not a novel issue, as it has been addressed in movies and publications in diverse fields [5,6], and was recently put in the spotlight of public debate as a consequence of privacy lawsuits against the big information technology corporations [7]. While the use of such personal big data for profiting purposes has been unveiled, its use for mental health purposes remain largely unknown. People share images, videos, and texts on their social media, showing how they behave and what they speak and think. These are the very tools used by mental health professionals to make their diagnosis. Evidently the issues displayed in social media are not the ones investigated in a mental health consultation, but they often overlap as users frequently post their intimate feelings, share their mood, and so on, online. Besides this, AI techniques have evolved to an extraordinary level, and their machine learning (ML) algorithms for verbal and nonverbal language analyses of individuals has evolved likewise [8,9]. A study published in 2020 used language and images posted to Facebook to identify signs associated with psychiatric illness [10]. A ML algorithm was fed with 3,404,959 Facebook messages and 142,390 images across 223 participants with schizophrenia spectrum disorder or mood disorders and healthy volunteers. All data prior to the first psychiatric hospitalization was uploaded to minimize the potential confounds on social media activity of medications, hospitalizations, and relapses, and receiving a formal psychiatric diagnosis. The algorithm was able to differentiate the diagnosis using Facebook activity alone over a year in advance of the first-episode hospitalization, with areas under the curve (AUCs) varying between 0.72-0.77.

Regarding this “visibility” of severe mental disorders, in more clinical/research settings, video diagnosis frameworks have also been tested, with encouraging results. Researchers have found that neuromotor precursors of schizophrenia, for instance, can be traced back to childhood [11]. Accordingly, an analysis of brief videotape footage of children eating lunch suggested that observed movement anomalies were able to discriminate among those children who later developed schizophrenia and those who did not [12]. More recently, verbal language features extracted from video and audio recordings were shown to be important early signs of psychotic illness [13-17]. These features include discourse coherence, syntactic complexity, speech content poverty, metaphorical language, and language structural connectedness [13-17]. Bedi et al [13], for instance, showed that discourse coherence was significantly correlated with subclinical psychotic symptoms. Though derived from a small sample of individuals with at-risk mental states (ARMSs) for psychosis, their model could predict with 100% accuracy progression from the risk state to schizophrenia. This finding of lower speech connectedness in at-risk individuals was also replicated by Spencer et al [17], who used speech graphs in their analyses, another technique to assess syntactic complexity and speech content poverty. These features are commonly referred to as natural language processing and, in larger samples, were used to obtain AUCs as high as 0.88 for predicting which at-risk individuals will develop a psychotic disorder in the future. For individuals at first-episode psychosis, AUCs of up to 0.92 were obtained to predict who would receive the diagnosis of schizophrenia 6 months in advance [16]. Audio features such as pauses [18] and nonverbal behavior such as gestures [19] and movement in general [20] were also seen to be discriminative between healthy and ARMSs. Besides serious mental disorders [21], AI frameworks have also been developed to detect and classify other mental disorders, as shown by numerous publications and challenges to establish an accurate depression estimation from audio, video, and text information [22,23].

Thus, the possibility to “see” mental disorders is, per se, an innovative technology. It could increase access to mental health care and allow for prevention, early diagnosis, and treatment, as in the cases of the illnesses cited in the beginning of this text. It could also aid clinicians in diagnosing, following-up, and prognosing their patients in their daily practice. This would greatly improve the outcome and quality of life for those afflicted with a mental illness. However, one issue distinguishes mental disorders from other illnesses, making the idea of “seeing” them less desirable. This issue is stigma.

## Stigma of Mental Illness

Mental illness carries a great deal of social stigma, which most physical illnesses do not [24]. Prejudice and discrimination toward people with mental disorders is historical, existing from before the birth of psychiatry [25,26]. Unfortunately, despite the great advances seen in psychiatry in the last decades, stigma still persists in several forms [27]. Its causes are many, but as mental disorders generate behavioral changes, they often tend to be judged by the public as a moral act of the patient. If one conceives mental illness as a strictly biological disease, with genetic causes, for instance, fear and feelings of insecurity arise toward the mentally ill, as if the person would not have control of their actions [28]. If mental illness is conceived as a sole psychosocial issue, the patients are to blame for their depressive symptoms, anxiety, and so on. The situation is worse for the group of severe mental disorders. Hallucinations and delusions seen in schizophrenia symbolize a mismatch of people with the disorder with the public’s common experience, generating fear, perceived danger, and the desire for social distancing [29-31].

As such, stigma constitutes a major treatment barrier for those with mental disorders, hampering preventative initiatives and substantially worsening outcomes. This barrier appears before treatment has begun (low mental health literacy, stereotypes endorsement, and diagnosis delay and denial) and continues thereafter (self-stigma, low treatment adherence, diagnosis disclosure) [32]. Stigma also contaminates those that surround the mentally ill, including family members, friends, and health professionals. Reproducing the prejudice seen in other illnesses such as AIDS and leprosy in the past, mental disorders are often faced as if they were contagious [33]. This worsens discrimination and social isolation as even close people tend to stay away from those with the diagnosis—something called courtesy stigma [34,35]. Due to this multifaceted burden of stigma, revealing that someone may have a depressive disorder, or that someone may be at risk of having schizophrenia in the future, is different from pointing out that someone should seek a doctor because of a suspected thyroid or skin cancer [36].

To make the stigma issue worse, there is the problem of false positives [37]. There is an ongoing debate on the accuracy of one of the most studied preventive paradigms in psychiatry, namely, the ARMS for psychosis concept (or clinical high risk for psychosis) [38]. Researchers are trying to enhance the accuracy of the ARMS criteria, as studies showed that most individuals that fall into the criteria (76%) do not develop a psychotic disorder at all [39]. In other words, the use of ARMS criteria alone generates a large number of false positives. The use of language to classify ARMS individuals who will develop a future disorder can potentially predict up to 80%-90% of cases, as seen in some studies—though with small samples. This accuracy is similar to that of a pap smear to screen for cervical cancer, eliciting a false-positive rate of around 20% [40]. However, while pap smears are routinely used as an important preventive public health strategy and concerns are directed toward improving the false-*negative* ratio [41], “seeing” severe mental disorders through language analysis would still be a concern. Even though language frameworks can substantially reduce the number of false positives, the great stigma related to the condition and the uncertainty regarding intervention at this phase still hinder the implementation of preventive strategies. As such, the number of prevented cases must be weighed against the number of individuals harmed by being misdiagnosed as being at risk [42].

## Bioethical Issues

Given that mental disorders might be now “visible” to AI algorithms that analyze communication, and that there is a stigma related to these disorders, one must consider the bioethical implications. The four main principles of bioethics are (1) beneficence, (2) nonmaleficence, (3) autonomy, and (4) justice [43].

Beneficence is the principle that guides physicians to act for the benefit of patients. It also implies several other actions beyond the usual patient-doctor relationship in a clinic, such as rescuing persons in danger, removing conditions that will cause harm, and helping individuals with a disability. Beneficence is a positive concept in the sense that one has to be active, to propose actions and intervene. Nonmaleficence, on the other hand, concerns the obligation physicians have to not harm their patients, not worsen their health, not incapacitate, not cause suffering, and not deprive others of the goods of life. In practice this implicates the weighting of benefits against burdens of all interventions and treatments and in considering not to act [43]. Autonomy asserts that every person has the power to make rational decisions and moral choices, and everybody should be allowed to exercise their capacity for self-determination. The principle of autonomy branches out into three other important principles [43]:

1. Informed consent: patients must receive full disclosure and comprehend the disclosure to voluntarily agree to a medical procedure or research.
2. Truth-telling: a vital component in a physician-patient relationship, as autonomous patients have the right to know their diagnosis but also the option to forgo this disclosure.
3. Confidentiality: physicians are obliged not to disclose any confidential information given by patients to another party without their consent.

Justice is the fourth ethical principle. It encompasses the need for a fair, equitable and appropriate treatment of persons. This principle may encompass microsettings, such as adequately treating individuals in an emergency service, as well as macrosettings involving health care policies.

That being said, the use of language detection algorithms for mental health purposes may pose some bioethical dilemmas, and the use of such tools must still be approached with caution. The role of medical advice and diagnosis in one’s disease trajectory is relevant, and the early detection of mental illness can enable health care practitioners to intervene and avoid negative outcomes. On the other hand, the attribution of labels can also increase the chances of self-stigma [42]. Social stigma is a great burden for people with mental disorders and is especially associated with the psychiatric diagnosis (ie, labels) [29], generating poorer outcomes and other negative consequences [44]. This is especially important considering the high rate of false positives among the previously cited ARMS condition [39]. That is, individuals (wrongly) classified as ARMS but who would never develop a mental disorder. Using the ARMS paradigm indiscriminately to diagnose individuals without properly demystifying this information and destigmatizing mental illnesses would constitute a threat to the nonmaleficence principle [42]. Besides, it would also be potentially paternalistic, harming autonomy.

To understand the biological pathways toward psychosis and to develop new treatments, research efforts are being directed to the enhancement of the predictive power of the ARMS concept [45]. This is being done by investigating biological markers or by using ML algorithms. In this sense, the use of AI for natural language processing has produced encouraging results, with a diagnostic accuracy similar or superior to other classifiers used in medicine [14,15]. However, if stigma is not addressed in a comprehensive way, no matter how few false positives there are, they will still be a concern regarding nonmaleficence. Moreover, the use of such algorithms to interpret language data also poses a threat to nonmaleficence and to autonomy, more specifically concerning the lack of comprehension of certain information given by such algorithms, which we depict below.

## Interpretability and Validity of Algorithms

The issue that some ML models are impossible to interpret has recently gained a growing interest [46]. There’s an ongoing discussion on the repercussions of such algorithms for high-stakes decisions. Such models are called black box models, for their operation with the inputted variables is not completely observable. They are known to learn from subtle metadata, and this may carry the risk of hidden bias (eg, the Clever Hans problem) [47].

Exemplifying this, in 2018, a study aimed to investigate the generalizability across sites of a deep learning model to detect pneumonia in chest radiographs [48]. At first, the model performed very well in distinguishing high-risk patients from non–high-risk patients. However, upon external validation, the performance plummeted. The AI model was not learning from clinically relevant information from the images. Instead, it was learning and basing its decisions on hardware-related metadata tied to a specific x-ray machine. This machine was being used in an intensive care unit (ICU) to image mostly high-risk individuals [49]. That is, the algorithm would attribute a high-risk classification to most images coming from that ICU’s x-ray, instead of using clinical data from the x-rays themselves to make decisions. Several scholars have discussed explainability as a major problem for the use of AI in health care [50].

For some computational problems, it is hard to associate meaningful parameters with individual variables. For instance, in images examined by computer vision, each pixel is meaningless without context, while the full set of pixels taken together contain local (eg, pixels that together form a smile) and global (eg, sources of light inferred from shadow directions) characteristics. Complex models make use of several heuristics to capture abstract notions according to each application. Concepts such as objects in pictures and seasonality in time series are encoded and distributed across different structures within the model. Therefore, simple descriptions such as “anxiety increases as stress increases” are rarely possible, contrary to what happens in familiar regression methods. Since multiple conditional dependencies preclude direct statements about results, additional analytical and experimental steps are required for the interpretation of complex models [51].

In short, it is not enough only to enhance predictive power and avoid false positives but also to understand the real-world underpinnings of black box algorithms [49]. Both machine statements and human statements are congruent with a given ground truth [50]. Taking the above example, we have two statements, accordingly. First, a specific characteristic of some given x-rays is associated with a higher risk for pneumonia (machine statement). Second, we should prioritize patients with those x-rays, as they are at higher odds of having pneumonia according to the ML model (human statement). Both statements are equally used for decision-making. Nevertheless, human models are often based on causality as an aim for understanding underlying mechanisms and for intervention. While the correlation is accepted as a basis for decisions, it is viewed as an intermediate step. For instance, *why* are those specific patients at higher risk of having pneumonia? We should investigate their characteristics to understand the higher risk of pneumonia and to develop a specific antibiotic. On the other hand, ML algorithms are typically based on probabilistic models and provide only a crude basis for further establishing causal relationships. Upon opening the black box, the relationship between that specific set of x-rays and pneumonia was due to a given x-ray machine located in an ICU service that was working on many more cases of pneumonia than the other machines. That is, ML models offer important decision-making tools, namely, prioritizing those individuals. However, further investigations beyond the simple association should be conducted, opening the black box and addressing physiopathological explainability and causability.

Decisions in health care imply liability, including legal and financial repercussions. Therefore, each decision must be logically reasoned with explainable evidence [49]. AI models might be insightful for scientists, but they should also be sufficiently clear and explainable for end users to support their decisions [52]. Otherwise, it could constitute a threat to the patient’s autonomy. Accordingly, traditional algorithms must handle sources of information in an interpretable manner, such as the GRACE score for acute coronary syndrome and the Sequential Organ Failure Assessment score for organ failure in ICUs [53,54]. They map higher probabilities of a bad outcome to signs of severity (eg, abnormal values in biological markers). Clinical support decision systems based on opaque (“black-box”) algorithms must, as such, provide a clear rationale to be useful for practitioners. Besides carrying hidden bias, the use of opaque algorithms leads to a defensive medical practice. When no underlying rationale is presented [55], physicians tend to agree with the machine to avoid liability. On the other hand, interpretable outputs will help practitioners to treat their patients with fewer overlooked findings and misled predictions.

Post hoc techniques of analysis (local interpretable model-agnostic explanations, Shapley Additive Explanations, multilingual unsupervised and supervised embeddings, etc) are an option when model parameters are numerous and computational processes go beyond elementary functions and operators [51]. Specifically regarding language data, researchers should treat findings from computer based evaluations as they do with traditional indicators. It is crucial, for instance, to have representative data as a basis for normative curves for each proposed behavioral marker. How does it develop through ages; how does it change according to gender or ethnicity; what are the effects of social factors such as socioeconomic status, educational level, neighborhood, or exposure to urban violence? Before jumping to the conclusion on the relationship of some behavioral marker to a pathological factor, we first need to map and understand normative variability across cultures, languages, and countries [56,57]. For example, language structural connectedness that diminishes according to negative symptom severity under psychosis [16] also increases during typical development, being tightly associated with educational levels. Years of formal education are more important to explain this developmental trajectory than years of age to the point that illiterate adults narrate their stories with a structural pattern similar to preschool children [58].

After gaining insights on potential pathological markers and mapping on pathological confounding factors, we still need to discuss potential public policies that protect the individual rights to not be evaluated or even judged without consent.

## Legislation and Data Privacy

Otto Hahn won the Nobel Prize for discovering nuclear fission in 1939 and allowing nuclear reactors to produce enormous amounts of energy [59]. However, he is a controversial historical figure once his discovery also allowed the building of the World War II atomic bombs. Arthur W Galston studied the effects of 2,3,5-triiodobenzoic acid on the growth and flowering of soybeans. Later the military developed it into Agent Orange and used it in the Vietnam War as a chemical weapon. This led Galston to become a bioethicist and give talks on the misuse of science [60]. Likewise, internet, smartphones, social media, and search mechanisms revolutionized our relationship with knowledge and with each other as humans. However, unethical misuse of big data to control one’s exposure to information, to stimulate consumerism, and to capture someone’s time for profiting purposes are the proxies of such inventions’ perversion [61]. The number of discoveries—either scientific or not—that got misused by third parties other than their inventor is countless. It is easy to figure out how technologies that address mental status through language can follow the same way.

Automated analysis of free speech, for instance, can establish thought disorder indexes based on what someone says or writes [13]. Additionally, these indexes can predict future serious mental disorders like schizophrenia. That is, the data fed into the analysis can be extracted from written text from books, transcripts, or other data sets that are available to the public (eg, social media or personal blogs). This raises the possibility of malicious use, given the online availability of people’s written data on the internet. Another example of the possible use of available information to infer the mental status of individuals is the Facebook study mentioned at the beginning. The algorithm could predict a future and severe mental disorder with a 72%-77% level of certainty. All these works importantly advance science and provide the perspective of useful tools to be used by clinicians and policy makers. Additionally these findings are developed in environments strictly guided by ethical standards given by ethics committees and supervised by the scientific community. However, the problem is not related to these regulated settings but when the invention goes beyond scientists.

Likewise, ethical boards review and regulate scientific studies and health professionals’ practice; apart from these settings, the law exists to oversee and penalize irregular use of big data [62]. As such, there is now a growing concern about digital privacy, especially after the awareness raised by the lawsuits against big tech claiming too much power over people’s lives and personal information a few years ago. This has led to an increase in legislation to regulate access and use of personal information, especially that which is somewhat publicly available in online social networks. However, there is always the risk of a legal gap as cutting-edge inventions are temporally ahead of legislation protecting them from misuse. This is especially evident today as new ML algorithms and technologies arise with increasing frequency. This can potentially foster discrimination of individuals with mental disorders in countries where such a gap is not covered by personal information privacy protection legislation. For instance, allowing the misuse of such technologies in job interviews, academic interviews, and so on, to dismiss people from the selection process based on preconceived ideas about mental disorders.

## Conclusion

Summarizing, new technologies derived from AI have the potential to “see” mental disorders by someone’s behavior and discourse. These technologies per se would greatly help in early detection and disease outcome. However, the historical and enduring stigma attached to mental disorders hampers the use of such tools. Fighting prejudice and discrimination related to mental disorders should constitute future directions so that stigma does not constitute a barrier for the use of these innovative technologies. Moreover, to comply with nonmaleficence and avoid the stigma, these technologies also need to have low rates of false positives in predicting someone’s possibility of future mental illness. There is a further risk that these ML algorithms turn into black box models. This hidden bias problem could potentially harm the patients’ autonomy and disclosure. So, it is necessary to clearly describe the algorithm, to use post hoc interpretation methods, and to conduct bias-checking procedures. Additionally, because of stigma and due to the high online availability of personal information on an individual’s verbal and nonverbal language, information derived from the algorithms carries the risk of being misused, such as to discriminate against individuals because of their mental health status. In this sense, awareness should be raised in regulating the use of these technologies in real-world settings. There is a challenge for legislators to catch-up with the ever-renovating new technologies and algorithms designed to decipher human behavior to prevent these inventions from being misused.

## Abbreviations

AI: artificial intelligence
ARMS: at-risk mental state
AUC: area under the curve
ICU: intensive care unit
ML: machine learning
